# Data from camera surveys identifying co-occurrence and occupancy linkages between fishers (*Pekania pennanti*), rodent prey, mesocarnivores, and larger predators in mixed-conifer forests

**DOI:** 10.1016/j.dib.2016.01.032

**Published:** 2016-01-28

**Authors:** Rick A. Sweitzer, Brett J. Furnas

**Affiliations:** a Great Basin Institute (GBI), 16750 Mount Rose Highway, Reno, NV 89511, USA; b California Department of Fish and Wildlife (CDFW), 601 Locust Street, Redding, CA 96001, USA

**Keywords:** Carnivores, Competition, Distribution, Foraging guild, Predation, Tree squirrels

## Abstract

These data provide additional information relevant to the frequency of fisher detections by camera traps, and single-season occupancy and local persistence of fishers in small patches of forest habitats detailed elsewhere, “Landscape Fuel Reduction, Forest Fire, and Biophysical Linkages to Local Habitat Use and Local Persistence of Fishers (*Pekania pennanti*) in Sierra Nevada Mixed-conifer Forests” [10]. The data provides insight on camera trap detections of 3 fisher predators (bobcat [*Lynx rufus*]). Coyote [*Canis latrans*], mountain lion [*Puma concolor*], 5 mesocarnivores in the same foraging guild as fishers (gray fox [*Urocyon cinereoargenteus*]) ringtail [*Bassariscus astutus*], marten [*Martes americana*], striped skunk [*Mephitis mephitis*] spotted skunk [*Spilogale gracilis*], and 5 Sciuridae rodents that fishers consume as prey (Douglas squirrel [*Tamiasciurus douglasii*]), gray squirrel [*Sciurus griseus*], northern flying squirrel [*Glaucomys sabrinus*], long-eared chipmunk [*Neotamias quadrimaculatus*], California ground squirrel [*Spermophilus beecheyi*]. We used these data to identify basic patterns of co-occurrence with fishers, and to evaluate the relative importance of presence of competing mesocarnivores, rodent prey, and predators for fisher occupancy of small, 1 km^2^ grid cells of forest habitat.

## Specifications table

**Table:** 

Subject area	Biology
More specific subject area	Wildlife ecology, conservation biology
Type of data	Text, tables, figures
How data was acquired	Camera trap surveys
Data format	Tabular, plotted and analyzed
Experimental factors	Grid cells, presence/absence
Experimental features	Standardized camera trap surveys
Data source location	Sierra National Forest, California, USA
Data accessibility	Data in this article

## Value of the data

• These data provide new insights on how the distribution and habitat use of fishers is influenced by presence of multiple co-occurring carnivores and rodent prey in California, USA.
• These data indicated that fishers co-occurred with multiple species of rodent prey, multiple other mesocarnivores in the same foraging guild, and 3 larger predators that commonly attack and kill them.
• These data identified a positive association between fisher occupancy and presence of known prey of fishers, which was suggested previously but without supporting data [7].
• Mesocarnivores consume similar prey [12], and these data identified a negative association between fisher occupancy and presence of other mesocarnivores, indicative of interspecific competition.
• Previous research used presence records to predict the range of fishers [6], [7], [14], and data we provide on local occupancy of fishers with prey and competing mesocarnivores can improve models of their distribution in forest ecosystems.

## 1 Data

In this Data in Brief article we summarize camera trap detections of 3 fisher predators (bobcat, coyote, mountain lion), 5 mesocarnivores in the same foraging guild as fishers (gray fox, ringtail, American marten, striped skunk, spotted skunk), and 5 Sciuridae rodents that fishers prey on (Douglas squirrel, gray squirrel, northern flying squirrel, long-eared chipmunk, California ground squirrel) in the Sierra Nevada region of California, USA. These data identify basic patterns of co-occurrence of rodent prey and other carnivores with fishers, as well as how presence of these species influence fisher occupancy within small, 1-km^2^ patches of forest habitat in California, USA.

## 2 Experimental design, materials and methods

### 2.1 Study area

The overall research area was 1127 km^2^, and encompassed the non-wilderness region of the Bass Lake Ranger District in the Sierra NF, and a relatively small portion of Yosemite NP where camera trap surveys were completed between October 2007 and October 2014 [10]. The study area was centered in the California Wildlife Habitat Relations (CWHR) Sierran mixed-conifer forest habitat type (http://www.dfg.ca.gov/biogeodata/cwhr/wildlife_habitats.asp). Additional details on the diversity of trees and shrubs, and historic and current land use within the study area were provided elsewhere [9], [10], [11].

### 2.2 Camera trap surveys

We used a 1-km^2^ grid matrix overlain on the research area for organizing camera trap surveys. Motion sensing camera traps (Silent Image Professional, Rapidfire PC85; RECONYX Inc., Holmen, WI) were systematically deployed near the center of 1-km^2^ grid cells at the start of each of 7 camera survey years beginning around October 15 and ending the next year in early October. We placed camera traps within cells in the grid matrix by navigating to grid centers with a handheld Global Positioning System unit (Garmin model 60 CSx; Olathe, KS), and placing camera traps at the nearest position including one or more habitat elements known important for fishers [8]. Cameras were focused on the base and lower bole of bait trees, upon which we attached baits 1.1–1.5 m up from base, and applied scent lures as attractants. We used small pieces of venison (140–250 g) in a dark colored sock as meat bait for fishers, and 8–10 hard-shell pecans strung onto a length of wire and formed into a small ring as a nut bait for squirrels [8]. Scent lures were Hawbaker׳s Fisher Scent Lure (Fort Loudon, PA), Caven׳s “Gusto” scent lure (Minnesota Trapline Products, Pennock, MN), and peanut butter smeared on the nut ring, and we set all cameras to high trigger sensitivity, 3 pictures per trigger event on a 1 s interval, and no delay for images between trigger events [10].

### 2.3 Image interpretation and processing

**Fig. 1:**
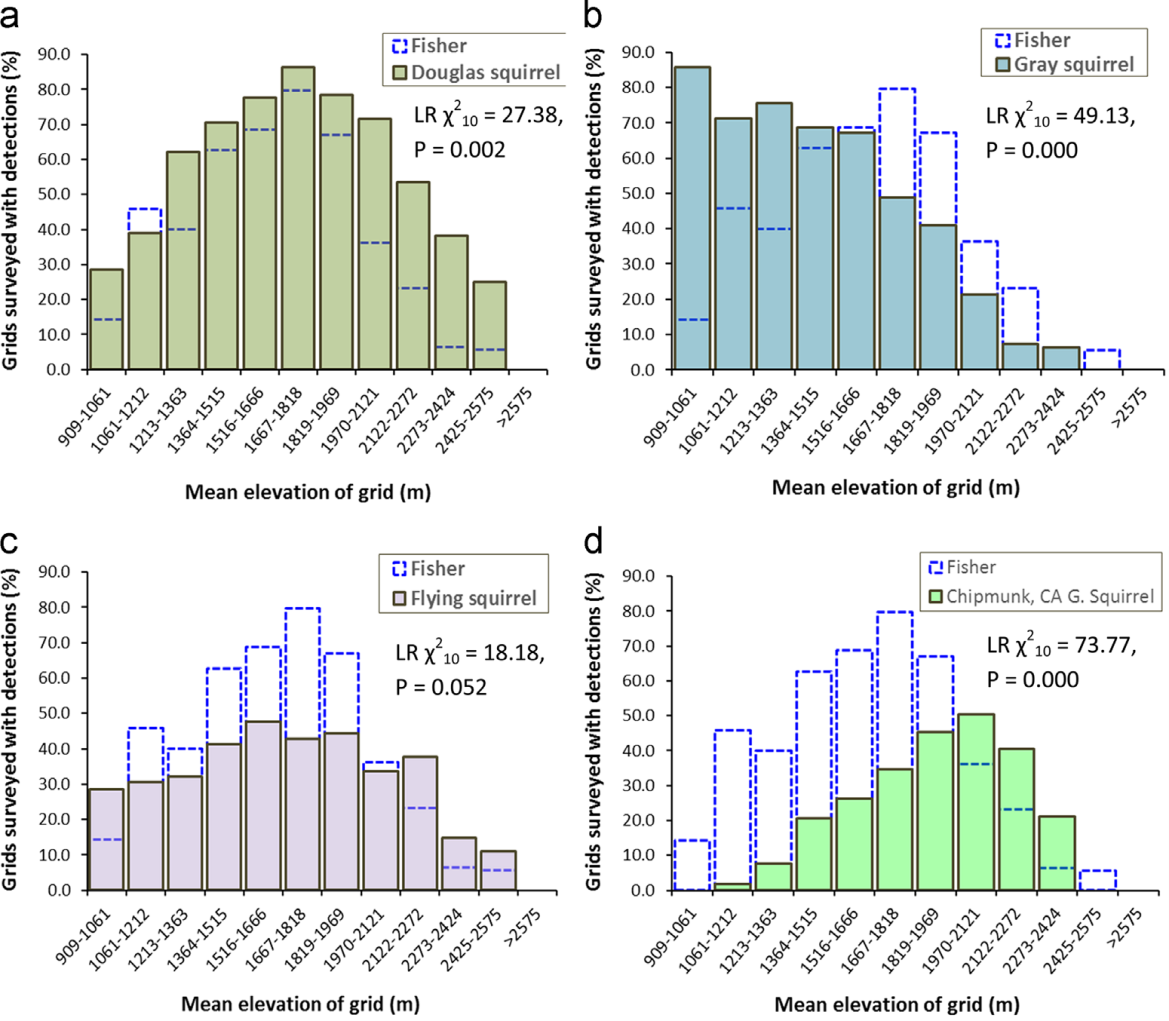
Distribution of camera trap detections within 1-km^2^ grid cells for 5 species of squirrels that fishers prey on in the research area. Douglas squirrel was the most commonly detected rodent prey (*n*=588) of all surveyed grids; (a), and this species completely overlapped with elevations where fishers were detected (Table 1). Gray squirrels were detected in 415 of the surveyed grids, and this species co-occurred with fishers between 909 m and 2424 m elevation (Table 1). Northern flying squirrels were detected in 328 of the surveyed grids, and occurred at all elevations where fishers were detected (Table 1). Long-eared chipmunk and California ground squirrel both hibernate during winter, which was reflected by fewer camera detections. Long-eared chipmunk or California ground squirrel were detected in 245 of surveyed grids and they co-occurred with fishers between 1364 and 2424 m elevation (Table 1). There was a high degree of elevation-based overlap between fishers and Sciuridae prey, but insight from loglinear *χ*^2^ analyses (LR *χ*^2^ metrics reported with each plot) suggested that frequencies of detections were different or trended different between fishers and all of the individual species or pairs of species.

**Fig. 2:**
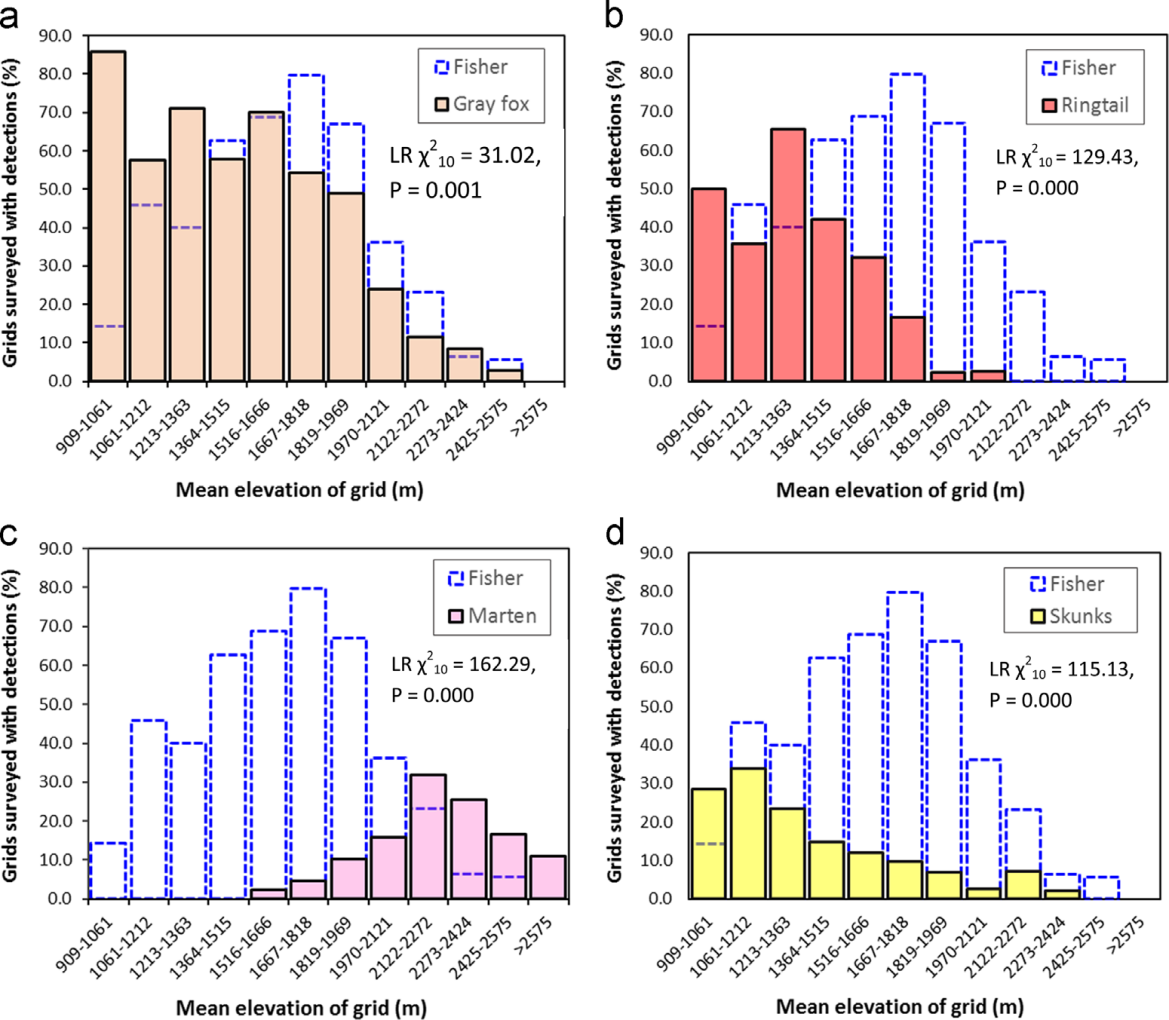
Distribution of camera trap detections within 1-km^2^ grid cells for 5 species of mesocarnivores. Gray fox was the most commonly detected (*n*=418), and this species was present at all elevations where fishers were detected (Table 1). Detections of ringtails were moderately common (*n*=200), and this species overlapped with fishers below 1666 m elevation (Table 1). Detections of American marten were less common (*n*=77), and marten overlapped with fishers primarily above 1970 m elevation (Table 1). Detections of striped and spotted skunks were infrequent (*n*=43, and *n*=61) surveyed grids, respectively, and skunks overlapped with fishers primarily below ~1969 m elevation (Table 1). Insight from loglinear *χ*^2^ analyses (LR *χ*^2^ metrics reported with each plot) included that the frequencies of detections were different between each mesocarnivore species and fisher.

**Fig. 3:**
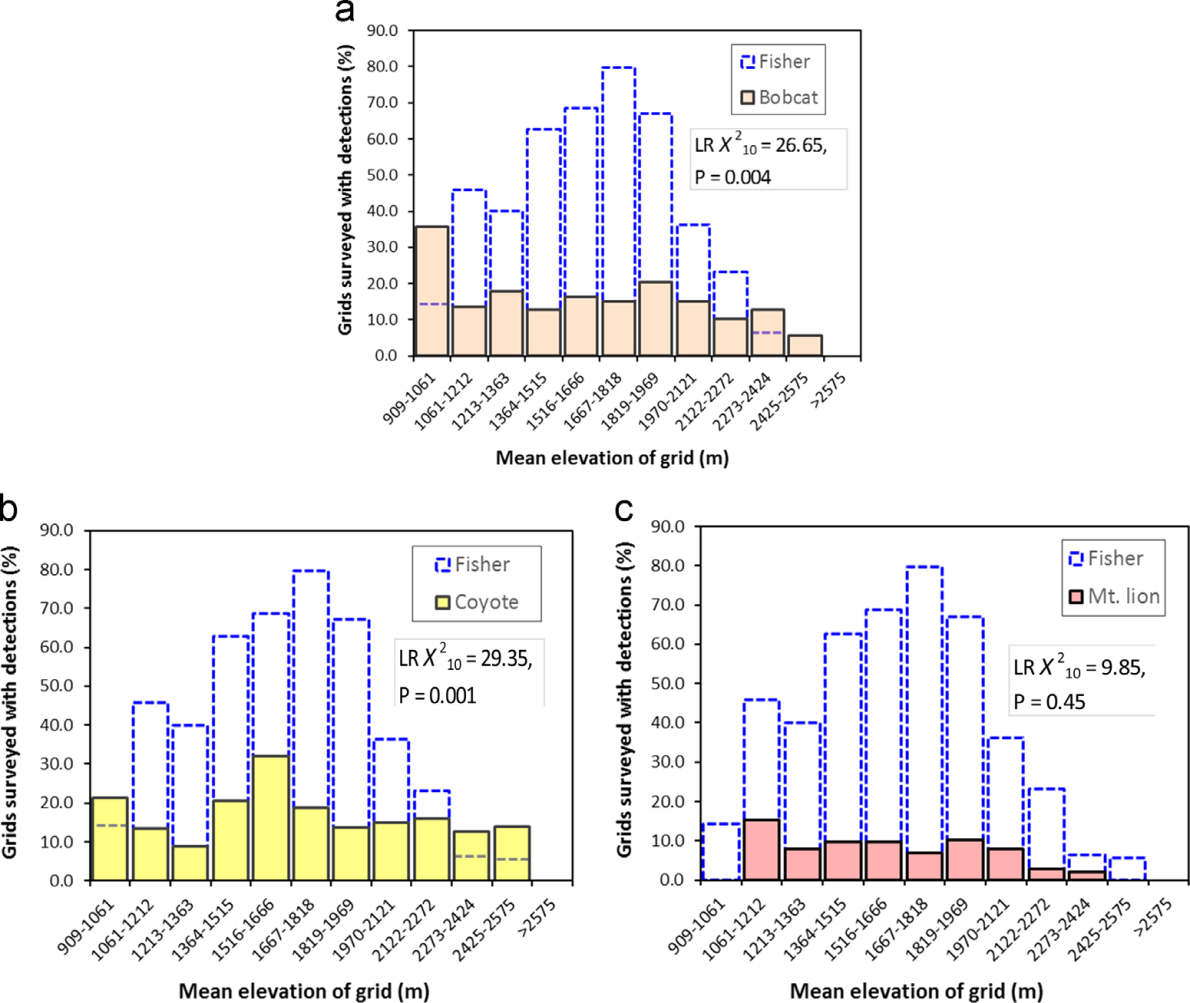
Distribution of camera trap detections within 1-km^2^ grid cells for bobcat (a), coyote (b), and mountain lion (c) overlain on fisher detections (bars with dashed lines). Bobcats were detected in 134 of the surveyed grids, and at all elevations where fishers were detected (Table 1). Coyotes were detected in 159 of the surveyed grids, and at all elevations where fishers were detected (Table 1). Mountain lions were detected in 69 (8%) of the surveyed grids, and overlapped with fishers from 1061 m to 2424 m elevation (Table 1). Insight from loglinear *χ*^2^ analyses (LR *χ*^2^ metrics reported with each plot) suggested that frequencies of detections were different between bobcat and fisher, and between coyote and fisher. Frequencies of detection were similar between mountain lion and fisher.

When we processed images from camera traps we assigned identity for each species and summarized data on detections to identify basic patterns of co-occurrence with fishers (Table 1). We represented co-occurrence of each species with fishers as the proportion of all camera trap survey stations where they were detected that overlapped with fisher detections from 909 m to 2707 m elevation (Fig. 1Fig. 2, Fig. 3). Each camera station was assigned an elevation based on the mean elevation for the 1-km^2^ grid [10]. For our assessment of general patterns of co-occurrence, we grouped camera traps into 12 bins (each bin spanned 151 m elevation), and created histograms representing the distribution of detections for each species that were plotted together with the distribution of fishers (Fig. 1Fig. 2, Fig. 3). Because we surveyed just 9 1-km^2^ grids with mean elevations≥2575 (bin 12), we combined species detections for bin 12 with bin 11. We used loglinear *χ*^2^ analyses to contrast detection frequencies between fishers and each species, or pair of species (e.g. long-eared chipmunks+California ground squirrels), and the statistical data were reported with the histograms (Fig. 1–3).

We developed 3 covariates from detections of other species at the camera traps. Metadata from images of bobcats, mountain lions, and coyotes were used to develop an index of the frequency of predator presence (pred) based on the number of 24 h calendar days with predator detections/effective camera days. Data on frequency of detection of 5 mesocarnivores in a similar foraging guild as fishers were included in the variable “compete” as an index of competition. We reviewed information on rodents consumed by fishers in the Sierra Nevada [13], and combined data on camera detections for them for the covariate “prey”, representing an index of prey availability in each 1-km^2^ survey grid.

### 2.4 Basic habitat and biophysical covariates

We developed local, cell-specific, biophysical covariates for use in analytical models of occupancy. We calculated the mean elevation (elev) for each surveyed cell, which was always included in occupancy analyses with its quadratic term (elev^2^). This covariate was standardized. Habitat covariates included an index of canopy cover based on the proportion of each cell with CWHR conifer and hardwood tree canopy closure classes M (40–59% canopy closure) or D (60–100% closure) (denMD; http://www.dfg.ca.gov/biogeodata/cwhr/wildlife_habitats.asp). We did not include covariates representing average tree size and slope because of their colinearity with forest cover and elevation.

### 2.5 Single-season occupancy model analyses

Occupancy represents the proportion of an area on which a species occurs [3], [4], and modeling can be used to estimate occupancy while accounting for heterogeneity in detection probability among survey sites [5]. We modeled single-season occupancy (ψ) and detection probability (p) as functions of covariates (*x*) and parameters (β) where p was defined as the probability of observing fisher during a survey period if it was present.

Single-season Occupancy Model

$$Detection:\;logit(p)=\beta_{p0}+\beta_{p1}x_{1}+\beta_{p2}x_{2}+$$

$$Occupancy:\;logit(\psi )=\beta_{y0}+\beta_{y1}x_{1}+\beta_{y2}x_{2}+$$

We created a detection history of whether a fisher was observed by a camera trap within each grid during each consecutive survey period after set-up or re-baiting for up to 5 8–10 day periods during a survey year, detailed elsewhere [10]. Models were solved by maximum likelihood estimation (MLE) via R statistical software (Version 3.0.1, www.r-project.org) using the *unmarked* package [2]. Single-season occupancy models were fit using the *occu* function, and we followed an information-theoretic approach for comparing models containing different combinations of covariates. We evaluated the top models with AIC weights summing to 0.95 [1]. We based decisions on which covariates were important predictors of detection probability, and occupancy on the relative AIC weights of the top models and the magnitude and variation of parameter estimates from these models.

**Fig. 4:**
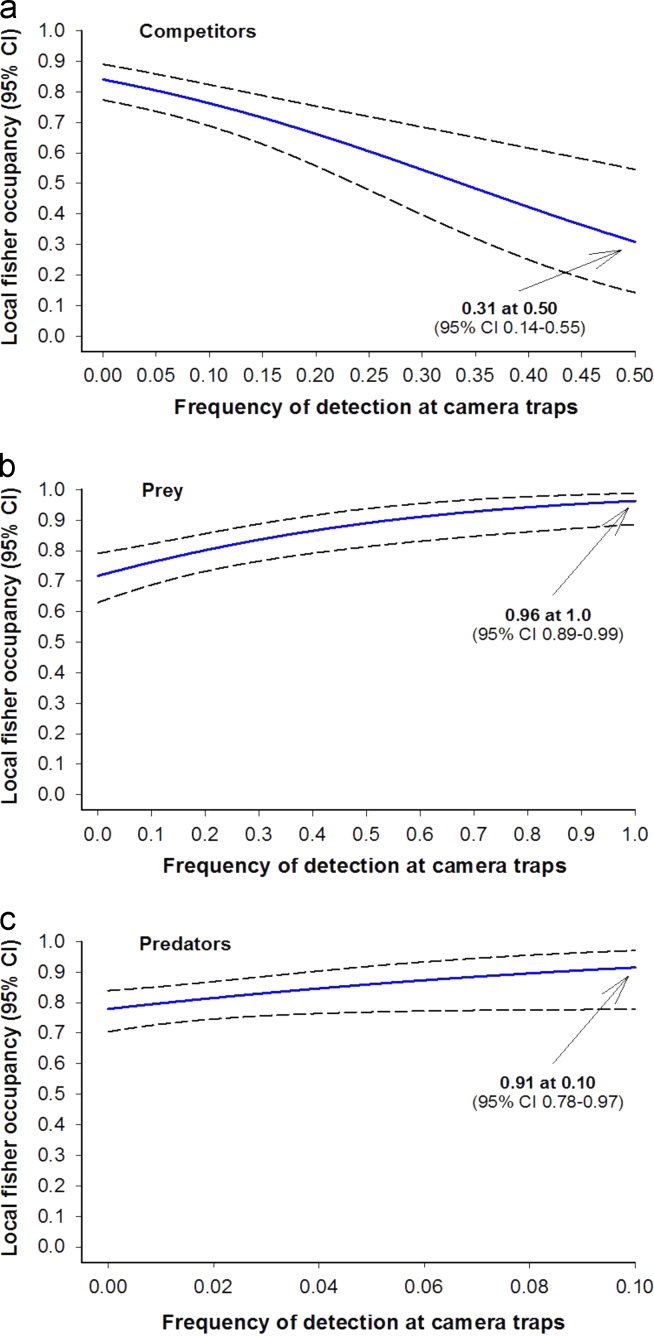
Single-season model illustrating the relationship between local fisher occupancy in 1-km^2^ grid cells and frequency of presence of competing mesocarnivores in the same foraging guild as fishers (compete; panel a), frequency of presence of rodent prey (panel b), and frequency of presence of 3 larger predators that attack and kill fishers in the study area (panel c). Fitted values were calculated assuming average values of elevation and canopy cover (e.g. denMD) from the 894 sites where surveys occurred. These data provide evidence for a negative association between local fisher occupancy and presence of other mesocarnivores, as well as indications for a strong positive association between fisher occupancy and presence of rodent prey. Due to the relatively wide 95% CIS, we considered that there was limited evidence for an association between fisher occupancy and presence of 3 large predators that kill them.

Covariates for potentially explaining detection probability included a dichotomous, 1st order Markov process reflecting whether a fisher was detected in the previous survey period in a season (auto.y); the number of effective camera days in a survey period divided by 10 (camdays), the proportion of CWHR medium and dense canopy closure classes in each grid (denMD), and a dichotomous variable representing whether the survey was conducted in summer (summer) instead of in fall to spring [10]. We fit all 16 combinations of these detection covariates in occupancy-intercept-only single-season models (e.g., logit(ψ)=β_ψ0_, logit(p)=β_p0_+β_p1_x_1_+β_p2_x_2_+…). Covariates deemed important in this step were included in the detection component of all subsequent models. Next, we evaluated the following occupancy covariates: compete, prey, pred, elev+elev^2^, and denMD. While always including the final detection covariates, we fit all 64 possible combinations of the occupancy covariates in single-season models. We evaluated these models to assess the importance of occupancy covariates and to identify a “best model” for estimation of detection and occupancy parameters (Table 2). We used parameter estimates from the best model (Table 3) to investigate potential linkages between local fisher occupancy and presence of competitors (Fig. 4a), presence of rodent prey (Fig. 4b), and presence of 3 larger predators (Fig. 4c).

## Figures and Tables

**Table 1:** Data on camera trap detections within 1-km^2^ grid cells for fishers, large predators, medium-sized carnivores (mesocarnivores), and Sciurid rodents (rodent prey) that fishers are known to consume in the Sierra Nevada region of California, USA. Camera traps were partitioned into 12 151 m elevation bins based on the mean elevation for each grid cell estimated from a 10 m digital elevation model for the research area. Data are from surveys completed from October 2007 to October 2013 in the Sierra National Forest and southern Yosemite National Park [10].

Large predators						Mesocarnivores					Rodent prey				
Bin no. and elevation range	No. grids surveyed	Fisher	Coyote	Bobcat	Mt. Lion	Ringtail	Gray fox	Marten^a^	Striped skunk	Spotted skunk	Douglas squirrel	Gray squirrel	Flying squirrel^a^	Chipmunk^a^	Ground squirrel^a^
1. 909–1061 m	14	2	3	5		7	12		4		4	12	4		
2. 1061–1363 m	59	27	8	8	9	21	34		18	2	23	42	18	1	
3. 1213–1363 m	90	36	8	16	7	59	64		13	8	56	68	29	5	2
4. 1364–1515 m	102	64	21	13	10	43	59		9	6	72	70	42	12	9
5. 1516–1666 m	134	92	43	22	13	43	94	3	8	8	104	90	64	22	13
6. 1667–1818 m	133	106	25	20	9	22	72	6	3	10	115	65	57	25	21
7. 1819–1969 m	88	59	12	18	9	2	43	9	2	4	69	36	39	20	20
8. 1970–2121 m	113	41	17	17	9	3	27	18	1	2	81	24	38	31	26
9. 2122–2272 m	69	16	11	7	2		8	22	2	3	37	5	26	16	12
10. 2273–2424 m	47	3	6	6	1		4	12	1		18	3	7	8	2
11. 2425–2575 m	36	2	5	2			1	6			9		4		
12.>1275 m	9							1							
															
Totals	894	448	159	134	69	200	418	77	61	43	588	415	328	140	105

a Species names are American marten, northern flying squirrel, long-eared chipmunk, and California ground squirrel respectively.

**Table 2:** Candidate models for single-season occupancy for camera trap surveys and fisher detections in the Bass Lake District, Sierra National Forest, California, USA from October 2007 to October 2014.

Model, covariates	K^a^	AIC	ΔAIC	AICwt	Cumulative AICwt
*Detection; reviewed by Sweitzer et al.*[10]					
*Occupancy*					
compete+pred+prey+elev+I(elev^2^)+ denMD^b^	12	2197.02	0.00	0.76	0.76
compete+prey+elev+I(elev^2^)+ denMD	11	2199.68	2.66	0.20	0.96
compete+pred+prey+ elev+I(elev^2^)	11	2203.34	6.32	0.03	0.99
compete+prey+ elev+I(elev^2^)	10	2205.76	8.74	0.01	1.00
compete+pred+elev+I(elev^2^)+ denMD	11	2213.83	16.82	0.00	1.00
compete+elev+I(elev^2^)+ denMD	10	2214.87	17.86	0.00	1.00
pred+prey+elev+I(elev^2^)+ denMD	11	2215.23	18.21	0.00	1.00
prey+elev+I(elev^2^)+ denMD	10	2216.24	19.22	0.00	1.00
compete+pred+elev+I(elev^2^)	10	2221.20	24.18	0.00	1.00
compete+elev+I(elev^2^)	9	2221.93	24.91	0.00	1.00
pred+prey+ elev+I(elev^2^)	10	2223.36	26.34	0.00	1.00
prey+ elev+I(elev^2^)	9	2224.15	27.14	0.00	1.00
pred+elev+I(elev^2^)+ denMD	10	2226.99	29.98	0.00	1.00
elev+I(elev^2^)+ denMD	9	2227.37	30.36	0.00	1.00
pred+elev+I(elev^2^)	9	2235.29	38.27	0.00	1.00
elev+I(elev^2^)	8	2235.49	38.47	0.00	1.00
compete+pred+prey+denMD	10	2319.35	122.33	0.00	1.00
compete+prey+denMD	9	2328.09	131.07	0.00	1.00
compete+pred+prey	9	2334.30	137.28	0.00	1.00
compete+prey	8	2340.81	143.80	0.00	1.00
compete+pred+denMD	9	2345.27	148.25	0.00	1.00
pred+prey+denMD	9	2347.54	150.52	0.00	1.00
compete+denMD	8	2351.19	154.17	0.00	1.00
prey+denMD	8	2353.77	156.75	0.00	1.00
compete+pred	8	2362.75	165.73	0.00	1.00
pred+prey	8	2363.31	166.29	0.00	1.00
compete	7	2365.91	168.89	0.00	1.00
prey	7	2367.54	170.53	0.00	1.00
pred+denMD	8	2367.87	170.86	0.00	1.00
denMD	7	2372.75	175.74	0.00	1.00
pred	7	2385.93	188.91	0.00	1.00
Intercept Only	6	2388.52	191.50	0.00	1.00

a Number of parameters.

b This was the single best model of single-season fisher occupancy from our analyses.

**Table 3:** Parameter estimates for the best single-season model of fisher occupancy (*Ψ*=intercept+compete+prey+pred+elev+elev^2^+denMD; Table 1) from analyses of fisher detections within 1-km^2^ grid cells in the Sierra National Forest, California, USA.

Covariates, logit-scale	β	SE	95%CI L	95%CI U
Intercept	0.070	0.399	−0.712	0.852
Compete	−4.927	1.157	−7.195	−2.659
Pred	11.139	5.821	−0.270	22.548
Prey	2.313	0.654	1.032	3.595
Elev	−0.658	0.163	−0.978	−0.339
elev^ 2	−1.323	0.157	−1.630	−1.015
denMD	1.513	0.457	0.618	2.409
